# Impact of different harvest times on yield and quality of summer savory (*Satureja hortensis* L.) genotypes

**DOI:** 10.1371/journal.pone.0324133

**Published:** 2025-05-27

**Authors:** Ayse Ozlem Tursun

**Affiliations:** University of Malatya Turgut Ozal, Battalgazi Vocational High School, Battalgazi, Malatya, Turkey; University of Kashan, IRAN, ISLAMIC REPUBLIC OF

## Abstract

Summer savory (*Satureja hortensis* L.) is a plant of the Lamiaceae family that grows in various ecological regions of the world. It is one of the medicinal-aromatic plants with huge economic value. The objective of this study was to determine the yield and quality of eight different summer savory genotypes, harvested at three different developmental stages, i.e., beginning of blooming, 40–50% blooming and full bloom stage. A field experiment was conducted during 2021 and 2022, and parameters related to growth, yield, and essential oil content were recorded. The maximum plant height, fresh and dry herb yield, dry leaf yield and essential oil content were obtained at the full bloom stage. The highest fresh herb yield was attained by genotype G6 and G7during the first and second year, respectively, while genotype G7 produced the highest dry herb and dry leaf yield during both years. The highest essential oil yield was obtained from genotype G8 and G7 in the first and second year of the study, respectively. Carvacrol, γ-terpinene, α-terpinene and p-cymene were the most abundant essential oil components observed in summer savory genotypes harvested at different developmental stages. The frequently detected component in the essential oil was carvacrol, which was recorded at 40–50%blooming period. The results of the current study proved that the most suitable period for harvesting summer savory genotypes was full blooming period in terms of yield values, and 40–50% blooming period for extracting essential oil especially carvacrol.

## Introduction

Turkiye is rich in plant diversity and there are more than 12,000 plant taxa occurring naturally in the country [1]. Among these, medicinal and aromatic plants hold significance, and approximately 500 plant species are used for medicinal purposes in Turkiye [2,3]. Most of the commonly used medicinal plants belong to the Lamiaceae family, which is a large plant family comprising 245 genera and 7886 species [4]. This family includes several aromatic plant species and five genera (*Origanum* L., *Satureja* L., *Coridothymus* Rchb.f., *Thymbra* L. and *Thymus* L.) in the Lamiaceae family are collectively referred to as thyme [5]. Thyme stands out for its exceptional benefits, primarily the production of essential oils including thymol and carvacrol [6]. Species of the genus *Satureja* have significant economic and medicinal value due to their high essential oil content and uses in the food, cosmetics and pharmaceutical industry [7,8]. Summer savory (*Satureja hortensis* L.), is an annual herbaceous plant of the genus *Satureja* and is widely used in the food industry for food preservation [9,10] and in the medical and pharmaceutical industries for the production of dietary supplements and herbal medicine [11,12]. The essential oils of summer savory are a rich source of phytochemicals [13], and exhibit antioxidant [14], antimicrobial [15], antibacterial [16] and antifungal [17] effects. The most important essential oil components of the plant are p-cymene, thymol, carvacrol and γ-terpinene [18,19]. In addition, the extract of this plant also contains flavonoid, rosmarinic acid and caffeic acid derivatives. Among them, rosmarinic acid is present in high concentrations and is primarily responsible for the biological activity of the extracts [20,21].

The yield, essential oil content and quantitative composition of the plants are significantly influenced by the time of harvest as well as the ecological and climatic conditions [22,23]. The quality parameters of medicinal plants also depend on cultivar, geographical origin, and growth stages at the time of harvest [24]. Plant growth rate and essential oil content can be influenced by harvest timing and environmental factors [25], and essential oil yields can vary significantly between plants of same species and across different periods for the same genotype [26]. The developmental stage of the plant (ontogenesis) influences the biomass, yield and the composition of the essential oil [27]. Several studies have proved that the quantitative and qualitative composition of plant essential oils depend on the origin and location [28,29], the environmental conditions [30,31] and the developmental stages of the plant [32,33]. The yield, essential oil content and composition of some aromatic herbs can be influenced by harvest time and environmental and climatic conditions, as has been shown for oregano, marjoram and basil [22,23].

Previous studies have reported that the essential oil yield of medicinal plants can vary depending on growth stage and genotype of a plant. For example, in lavender, the essential oil content was found to be higher at the end of the blooming phase compared to the budding and full-bloom phases [34]. In *Origanum vulgare* L. ssp. *vulgare* and ssp. *hirtum*, the blooming phase was identified as the most productive period [35]. Similarly in *Thymus vulgaris*, the highest yield of dry and fresh herbage, along with the essential oil content was recorded at the beginning of the blooming period [36]. Meanwhile, for *Moldavian dragon* weed, the highest yield of fresh herbage and essential oil contents were observed at full-bloom [37].

Hence, cultivating the plant using appropriate cultivation techniques and meeting its ecological requirements is important. Moreover, it is important to determine the optimal harvest time to achieve the desired cultivation objectives particularly yield and especially essential oil content, which are the key quality criterion for medicinal and aromatic plants. Therefore, this study was conducted to evaluate the effects of harvesting different genotypes of summer savory at different time period under the ecological conditions of Malatya on the plant’s yield, quality and essential oil contents.

## Materials and methods

### Field description

Field studies were conducted in Malatya Turgut Ozal University Battalgazi/Malatya in 2021–2022 to determine the yield and yield components of the summer savory plant at different harvest times. The soil of the experimental area was sandy-loam with 38% sand, 22% silt, and 40% clay content. The pH of the soil was 8.6, with EC 0.3 dS m^-1^, the organic matter 2.6%, CaCO_3_ 36.1%, P_2_O_5_ 9.7 kg ha^-1^ and K_2_O 65.1 kg ha^-1^.

### Meteorological data

According to the climate data, the long-term average temperature (1929–2020) of the experimental area was 23.5 °C, while in 2021 it was 19.76 °C and in 2022 it was 18.0 °C. The amount of precipitation during the crop growing season was 73.2 mm in 2021 and 159.4 mm in 2022 (Table 1).

**Table 1:** Climate data during the summer savory growing season in 2021 and 2022.

	2021		2022	
Months	Rainfall (mm)	Average Temperature (°C)	Rainfall (mm)	Average Temperature (°C)
March	36.8	7.4	55.2	4.1
April	6.5	15.3	3	16.6
May	20.6	22	71.8	16.9
June	9.1	24.8	29.2	24.7
July	0.2	29.3	0.2	27.7
Total	73.2	--	159.4	--
Average	--	19.76	--	18.00

#### Experimental design.

The field was plowed by using chisel plow and the seedbed was prepared using disk harrow during both years. Plants were obtained from seeds of eight different summer savory (*S. hortensis* L.) genotypes provided by Isparta University of Applied Sciences. The accession numbers and origins of these genotypes are listed in Table 2. Since the seeds of summer savory are very small and require special germination conditions, they were sown on 24.03.2021 and 28.03.2022 under greenhouse conditions in a mixture of decomposed manure, horse manure and field soil (1:1:1). Once the seedlings reached a height of 10–15 cm, they were transplanted to the experimental plots on 29.05.2021 and 06.06.2022. The plants were transplanted with a spacing of 40 cm between rows and 25 cm between plants, while a distance of 0.5 m was maintained between the plots and 1 m between the blocks. Each plot consisted of four rows and the plot length was 4 m. Fertilizers N and P_2_O_5_ were applied at the time of planting at a rate of 25 kg ha^-1^ N and 50 kg ha^-1^ P_2_O_5_. The second dose of fertilizer equal to 25 kg N ha^-1^ was applied after one month of planting. In both the years, the weeds were removed manually using a hoe and by hand. Fields were irrigated with drip irrigation system. After reaching maturity, the middle two rows in each plot were harvested and used for obtaining crop yield (32 plants in each plot).

**Table 2:** Accession number and origin of the summer savory genotypes used in the experiments.

	Accession number	Origins
G1	W6 10932	Bulgaria
G2	PI 230265	Iranian
G3	ISBU1	Turkiye
G4	ISBU2	Turkiye
G5	PI 222258	Iranian
G6	PI 379540	North Macedonia
G7	ISBU3	Turkiye
G8	PI 226650	Iranian

The experiments were set up using randomized complete block design (RCBD) during with three replications during both the years. Summer savory plants were harvested at three different time periods: beginning of blooming (M1), 40–50% blooming (M2) and full blooming stage (M3). In each harvest period, parameters such as plant height (cm), fresh herb yield (kg ha^-1^), dry herb yield (kg ha^-1^), dry leaf yield (kg ha^-1^), essential oil ratio (%), essential oil yield (L ha^-1^) and changes in important components of essential oil were determined. For determining the dry weight and essential oil content, plant samples were dried in a shaded area (approximately 25 °C) for one week after harvest. The essential oil yield (L ha^-1^) was calculated by multiplying the essential oil content determined from each plot with the dry herb yield obtained from the unit area.

### Isolation of the essential oils

The essential oils were extracted using a 1-liter Clevenger distillation apparatus. Finely grounded 50 g of dried aerial parts were diluted with 500 ml of distilled water (1:10 w/v) under continuous heat for 3 h. After distillation, the apparatus was allowed to cool in order to carefully separate the essential oil from the water phase. The essential oil was dried over anhydrous sodium sulfate and stored in a dark glass bottle at 4 °C until analyses (Türkmen and Mert, 2020). The components of the essential oils from the plants were determined by the gas chromatographic method. The determination of essential oil components was carried out using a Thermo Scientific ISQ Single Quadrupole gas chromatograph under the following conditions, i.e., a TR-FAME MS column (5% phenyl polysilphenylene siloxane) that has an inner diameter of 0.25 mm × 60 m length, and a film that has 0.25 µm thickness. Helium (99.9% purity) was used as carrier gas at a flow rate of 1 mL min^-1^. The ionization energy of mass spectrometry was set to 70 eV, and the mass range (m/z) was 1.2–1200 amu. The scan mode was used for data acquisition. The MS transfer line temperature was set to 250°C, the MS ionization temperature to 220°C, and the injection port temperature to 220°C. The column temperature was initially set at 50°C and was increased to 220°C at a heating rate of 3°Cmin^-1^. The structure of each compound was identified from the mass spectra using the Xcalibur program (Wiley 9) [38].

### Statistical analyses

Data were subjected to statistical analysis. Generalized linear model (GLM) including one-way analysis of variance (ANOVA) was performed to evaluate the data in the studies for examining the effects of different harvest times of summer savory genotypes using IBM SPSS 25 statistical package program. The Duncan’s Multiple Range Test (DMRT) was used to compare the means and to determine the differences between treatments. The Origin pro 2025 package was used to generate graphs for plant height, fresh herb yield, dry herb yield, dry leaf yield, essential oil ratio, essential oil yield and change in important components in essential oil (carvacrol, γ-terpinene and α-terpinene).

## Results

**Fig 1:**
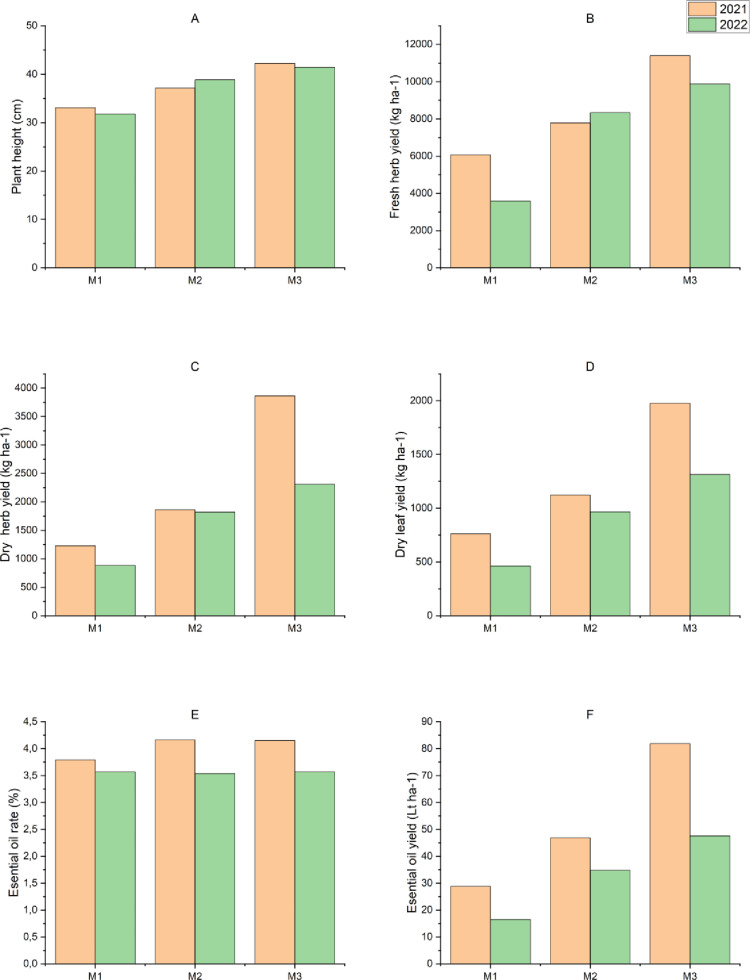
Effect of summer savory harvest time on yield and yield components.

**Fig 2:**
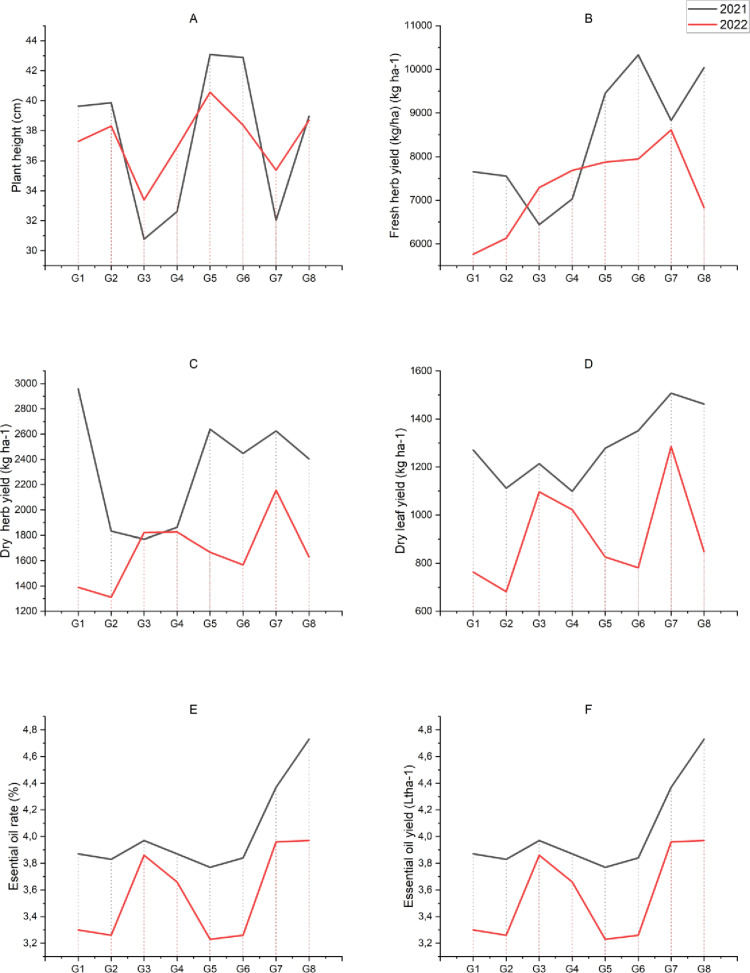
Effect of summer savory genotypes on plant yield and its components.

The results of the analysis of variance showed that data for plant height and fresh and dry herb yields of summer savory harvested at different developmental stages were statistically significant (p < 0.001) during 2021 and 2022. The genotypes (p < 0.001) and genotype×harvest time interaction (p < 0.01) showed a significant impact on all observed parameters except for dry herb yield in 2021 (Table 3, Fig 1A-F and Fig 2A, B, D, E, F).

**Table 3:** Variations in yield and yield components of summer savory genotypes at different harvest times.

	Plant height (cm)		Fresh herb (kg ha^-1^)		Dry herb (kg ha^-1^)		Dry leaf (kg ha^-1^)		Essential oil rate (%)		Essential oil yield (Lt ha^-1^)	
	2021	2022	2021	2022	2021	2022	2021	2022	2021	2022	2021	2022
G1 × M1	34.8 ± 0.9 gh	34.0 ± 1.2 ij	5468.7 ± 481.2 gh	3411.3 ± 212.8 i	1125.0 ± 109.9	866.7 ± 50.7 hi	646.00 ± 73.00 j	444.7 ± 22.3 jk	3.8 ± 0.1 e-i	3.4 ± 0.1	24.2 ± 2.8 hi	15.1 ± 0.6 hij
G1 × M2	38.6 ± 0.6 def	37.3 ± 0.8 efg	5885.7 ± 277.5 fgh	7144.3 ± 316.6 gh	1500.0 ± 17.9	1589.0 ± 11.0 ef	979.3 ± 27.3 hi	844.3 ± 11.3 gh	4.0 ± 0.0 d-h	3.2 ± 0.1	38.7 ± 1.3 fgh	27.2 ± 1.0 efg
G1 × M3	45.5 ± 0.6 b	40.5 ± 1.0 c	11604.3 ± 991.2 b	6733.7 ± 352.8 h	6250.0 ± 287.4	1711.3 ± 72.8 ef	2187.7 ± 136.3 ab	999.7 ± 33.3 efg	3.9 ± 0.2 d-h	3.3 ± 0.2	85.7 ± 8.6 bc	32.6 ± 0.6 de
G2 × M1	36.5 ± 0.3 efg	31.4 ± 0.3 kl	6390.0 ± 93.4 fgh	2989.0 ± 105.9 i	1142.0 ± 165.7	677.7 ± 44.7 i	761.7 ± 19.7 ij	333.3 ± 33.3 k	3.7 ± 0.1 e-i	3.6 ± 0.1	28.1 ± 1.5 ghi	11.8 ± 1.1 j
G2 × M2	39.6 ± 1.0 d	39.6 ± 0.1 cd	7073.0 ± 306.8 efg	7113.3 ± 462.5 gh	1750.0 ± 47.9	1455.7 ± 106.1 g	989.7 ± 45.6 hi	755.7 ± 58.8 hi	3.6 ± 0.4 f-i	3.1 ± 0.2	35.8 ± 5.1 f-i	23.1 ± 0.4 fgh
G2 × M3	43.5 ± 1.9 bc	43.9 ± 0.2 ab	9198.0 ± 329.9 cd	8288.7 ± 392.6 efg	2604.3 ± 130.5	1800.0 ± 115.5 ef	1583.3 ± 89.2 ef	955.7 ± 98.7 fg	4.2 ± 0.3 c-g	3.1 ± 0.3	65.6 ± 1.1 e	29.4 ± 1.3 def
G3 × M1	28.5 ± 0.3 k	28.6 ± 0.3 m	5115.0 ± 24.6 h	3333.0 ± 100.0 i	1109.0 ± 25.5	911.0 ± 11.0 hi	785.7 ± 18.7 ij	511.0 ± 11.0 jk	3.9 ± 0.1 d-i	3.8 ± 0.2	30.3 ± 0.5 f-i	19.4 ± 1.0 g-j
G3 × M2	30.9 ± 0.6 ijk	35.6 ± 0.6 hi	6031.3 ± 250.8 fgh	8578.0 ± 394.6 def	1604.3 ± 45.6	2055.7 ± 72.8 de	1031.3 ± 65.2 hi	1211.3 ± 29.4 cd	4.3 ± 0.2 b-f	4.0 ± 0.1	42.5 ± 5.4 fg	48.6 ± 1.6 c
G3 × M3	32.9 ± 0.1 hı	36.0 ± 0.3 gh	8177.0 ± 511.8 de	9966.7 ± 214.5 bcd	2594.0 ± 143.0	2500.0 ± 51.0 bc	1823.0 ± 99.4 cde	1566.3 ± 33.3 b	3.8 ± 0.1 d-i	3.8 ± 0.3	68.5 ± 2.6 de	59.1 ± 5.1 b
G4 × M1	29.2 ± 0.6 kj	31.8 ± 0.4 kl	5094.3 ± 380.0 h	3599.7 ± 328.3 i	1119.0 ± 90.7	922.3 ± 77.8 hi	660.7 ± 49.1 j	500.0 ± 38.7 jk	3.5 ± 0.1 hi	3.5 ± 0.2	23.1 ± 1.6 i	17.4 ± 1.0 hij
G4 × M2	32.0 ± 0.2 ıj	38.0 ± 0.5 ef	6104.3 ± 386.0 fgh	8355.7 ± 773.9 efg	1583.7 ± 75.1	1866.7 ± 171.2 ef	875.0 ± 17.9 ij	989.0 ± 95.0 efg	4.0 ± 0.2 d-h	3.7 ± 0.1	35.5 ± 1.7 f-i	36.4 ± 2.4 d
G4 × M3	36.6 ± 0.9 efg	40.9 ± 0.4 c	9896.0 ± 427.4 c	11099.7 ± 433.3 b	2885.3 ± 55.3	2689.0 ± 106.1 b	1760.3 ± 20.7 de	1578.0 ± 67.5 b	4.1 ± 0.3 d-h	3.8 ± 0.2	71.6 ± 4.4 de	59.5 ± 4.3 b
G5 × M1	38.3 ± 1.8 def	34.2 ± 0.2 i	7229.3 ± 239.7 ef	3989.0 ± 606.9 i	1406.3 ± 18.2	933.3 ± 134.8 hi	812.7 ± 18.2 ij	433.0 ± 57.7 jk	3.3 ± 0.2 i	3.3 ± 0.1	26.3 ± 1.4 hi	14.4 ± 1.9 ij
G5 × M2	42.5 ± 0.3 c	42.5 ± 0.2 b	8844.0 ± 221.5 cd	9344.7 ± 553.2 cde	1958.7 ± 90.7	1866.7 ± 134.8 ef	1135.7 ± 37.5 gh	900.0 ± 76.8 fgh	3.8 ± 0.1 d-i	3.2 ± 0.1	42.0 ± 1.2 fg	28.7 ± 2.0 def
G5 × M3	48.4 ± 0.8 a	44.9 ± 0.2 a	12281.3 ± 64.9 b	10288.7 ± 600.6 bc	4552.0 ± 1114.7	2200.0 ± 101.8 cd	1885.7 ± 27.7 cd	1144.3 ± 67.8 cde	4.3 ± 0.1 b-f	3.2 ± 0.2	79.8 ± 2.1 bcd	36.2 ± 4.1 d
G6 × M1	36.1 ± 10.2 fg	31.2 ± 0.8 kl	7329.3 ± 10.3 ef	3966.7 ± 562.0 i	1437.7 ± 36.1	833.3 ± 88.2 hi	833.7 ± 10.3 ij	422.3 ± 40.0 k	3.6 ± 0.1 ghi	3.3 ± 0.2	29.5 ± 1.4 f-i	13.7 ± 0.9 j
G6 × M2	42.6 ± 0.3 c	40.5 ± 0.2 c	9635.7 ± 478.0 cd	9469.0 ± 691.4 cde	2156.7 ± 78.7	1800.0 ± 157.5 ef	1156.3 ± 36.2 gh	877.7 ± 80.0 fgh	3.7 ± 0.3 e-i	3.0 ± 0.1	43.6 ± 4.1 f	26.2 ± 1.6 efg
G6 × M3	50.0 ± 1.3 a	43.5 ± 0.3 ab	14125.3 ± 853.3 a	10400.0 ± 400.5 bc	3750.0 ± 317.1	2066.7 ± 83.9 de	2062.7 ± 160.3 bc	1044.3 ± 58.8 def	4.3 ± 0.2 b-f	3.5 ± 0.3	87.3 ± 4.9 b	36.5 ± 3.7 d
G7 × M1	28.1 ± 0.2 k	30.7 ± 0.5 l	5489.7 ± 120.2 gh	4022.3 ± 80.0 i	1229.3 ± 27.3	1077.7 ± 40.0 h	823.3 ± 10.3 ij	611.3 ± 19.4 ij	3.9 ± 0.1 d-i	3.7 ± 0.2	31.7 ± 0.3 f-i	22.5 ± 2.2 f-i
G7 × M2	31.7 ± 0.5 ij	36.8 ± 0.2 fgh	8937.7 ± 281.8 cd	9166.7 ± 430.1 cde	2229.3 ± 136.5	2200.3 ± 120.2 cd	1354.3 ± 75.1 fg	1288.7 ± 80.2 c	4.8 ± 0.2 abc	4.3 ± 0.2	63.6 ± 1.2 e	56.0 ± 6.2 bc
G7 × M3	36.3 ± 1.3 efg	38.6 ± 0.3 de	12062.7 ± 55.6 b	12644.7 ± 895.4 a	4416.7 ± 875.3	3189.0 ± 214.5 a	2344.0 ± 82.6 a	1955.3 ± 112.8 a	4.4 ± 0.3 bc	3.9 ± 0.1	103.4 ± 7.7 a	75.8 ± 3.5 a
G8 × M1	33.2 ± 0.8 hi	32.6 ± 0.4 jk	6500.0 ± 219.0 fgh	3377.7 ± 251.0 i	1260.7 ± 102.4	844.7 ± 40.0 hi	760.7 ± 41.7 ij	433.3 ± 19.3 jk	4.9 ± 0.2 ab	4.0 ± 0.2	37.1 ± 3.6 f-i	17.4 ± 0.8 hij
G8 × M2	39.1 ± 0.6 de	40.4 ± 0.7 c	9739.7 ± 430.7 cd	7533.3 ± 221.9 fgh	2094.0 ± 190.9	1722.3 ± 39.4 ef	1458.3 ± 208.3 f	855.7 ± 29.4 gh	5.1 ± 0.1 a	3.8 ± 0.3	73.5 ± 10.2 cde	32.4 ± 2.5 de
G8 × M3	44.6 ± 1.6 bc	43.1 ± 0.5 b	13885.7 ± 713.7 a	9577.7 ± 135.3 cde	3854.3 ± 334.0	2322.0 ± 11.0 cd	2166.7 ± 133.3 ab	1255.7 ± 11.3 c	4.3 ± 0.4 b-e	4.1 ± 0.1	92.8 ± 7.9 ab	51.5 ± 1.2 bc
*F ration*	2.90^**^	4.78^***^	3.54^**^	3.85^**^	1.15^ns^	4.49^***^	3.44**	6.72***	2.72**	1.26ns	3.09**	6.90***

**p < 0.01 (moderately significant); ***p < 0.001 (highly significant); ns, non-significant.

Plants harvested at full bloom (M3) were taller compared to those harvested at the beginning of bloom (M1). As the summer savory plant continued to grow and continued to develop from the beginning of bloom to full bloom, and this resulted into an increase in their plant height. Among the genotypes, the highest plant height of 43.1 ± 1.6 cm and 40.6 ± 1.6 cm was observed in the G5 genotype of Iranian origin during 2021 and 2022, respectively. The lowest plant height of 30.8 ± 0.7 cm and 33.4 ± 1.2 cm was measured in the G3 genotype of Turkish origin during 2021 and 2022, respectively (Table 3, Fig 1A and Fig 2A). In the genotype×harvest time interaction, the highest plant height values for all genotypes were recorded at full blooming (M3), while the lowest values were recorded at the beginning of blooming (M1), during 2021 and 2022. The highest plant height was achieved by G6 genotype of North Macedonian origin (50.0 ± 1.3 cm) in 2021, while it was maximum by G5 genotype of Iranian origin (44.9 ± 0.2 cm) in 2022 at full blooming. In both the years, the lowest plant heights were recorded in the genotypes of Turkiye origin harvested at the beginning of blooming (M1) (Table 3, Fig 1A and Fig 2A).

The fresh and dry herbage yield of summer savory was higher at the beginning of blooming (M1) and full blooming (M3) in the first year, while in the second year; the fresh and dry herbage yields were higher when recorded at the 40–50% bloom (M2). In both the years, the lowest fresh and dry herb yield was obtained from the plants harvested at the beginning of blooming (M1), while the higher yield (fresh and dry herb) was obtained at full blooming (M3).The fresh herb yield was higher for G1, G2, G5, G6, G7 and G8 genotypes, while the yield of dry herbage was higher for all genotypes (except G3) in the first year, and the yields was decreased in the second year. The possible reason for this decrease in yield could be the difference in rainfall between the two years in the region where the trial was conducted. Among the genotypes, the highest fresh and dry yields were recorded for the genotypes G6 and G1 in 2021, while the genotype G7 produced the highest yield values in 2022. The lowest fresh and dry yields were obtained for G3 genotype of Turkish origin in the first year and G2 genotype of Iranian origin in the second year. In the genotype ×harvest time interaction, yields of fresh and dry herbage were higher for all genotypes at the beginning of blooming (M1) in the first year compared to the second year. When harvesting was performed at the 40–50% blooming stage (M2), the yield of fresh herbage was higher for genotypes G6 and G8 in 2021 compared to 2022 where genotypes G2 and G5 produced the higher yield of dry herbage. At the full blooming stage, the dry herb yield was higher in the first year than in the second year for all genotypes except G3, G4 and G7.

For all summer savory genotypes, the highest fresh and dry herb yields were obtained at the full blooming period in both the years. The highest yield in the first year was attained by genotype G6, while the lowest yield was attained by genotype G3. In 2021, the highest dry herb yield was achieved at full bloom (M3), and the highest dry herb yield was observed in genotype G1. In 2022, the highest yield of fresh and dry herb was obtained with genotype G7 (Table 3, Fig 1B, C and Fig 2B, C).

Based on the statistical analyses, the summer savory genotypes were significantly (p < 0.001) different in terms of dry leaf yield, essential oil content and essential oil yield (except for essential oil content in 2022), while in the genotype×harvest time interaction, a significant effect (p < 0.001 and p < 0. 01) was found in all values except for the 2022 essential oil content (Table 3, Fig 1D, E, F, Fig 2D, E, F).

The plants harvested at the beginning of blooming (M1) produced lower dry leaf yield than those harvested at full blooming stage (M3). The continued growth of the plant led to an increase in dry leaf yield. The highest dry leaf yield in both the years was recorded in genotype G7 among all genotypes, while the lowest dry leaf yield was achieved by genotype G4 of Turkish origin in 2021 and genotype G2 of Iranian origin in 2022. In the interaction between genotype×harvest time, a higher dry leaf yield was obtained in the first year than in the second year, both at the harvest times and among the genotypes (except for the G3 and G4 genotypes at the second cutting). The lowest values were obtained at the beginning of blooming and the highest dry leaf yields were obtained when the plants had reached full blooming. In 2021–2022, the highest dry leaf yield was obtained from the G7 genotype in the full bloom (M3) period.

The essential oil ratio and yield of the summer savory genotypes were the lowest when harvested at the beginning of blooming (M1) and these values reached the highest level when harvest was done at the full bloom period (M3). As the plants were closer to maturity, the essential oil (ratio and yield) of the plants were increased significantly. In addition, more essential oil was obtained in the first year compared to the second year. Among the genotypes, the highest essential oil ratio was obtained from the G8 genotype in both the years. While the essential oil yield was obtained from the G8 genotype in 2021, it was obtained from G7 in 2022. In the genotype×harvest time interaction, the highest essential oil yield was obtained from all genotypes at the full bloom period (M3) (Table 3, Fig 1E, F and Fig 2E, F).

**Fig 3:**
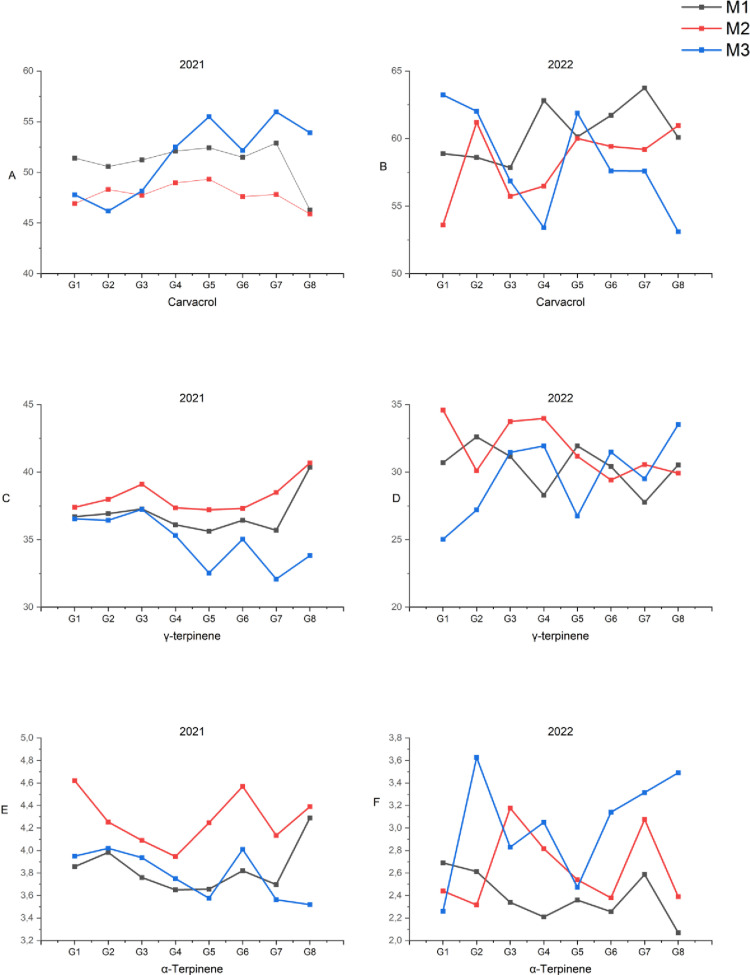
Changes in major essential oil components across summer savory genotypes over the years.

**Fig 4:**
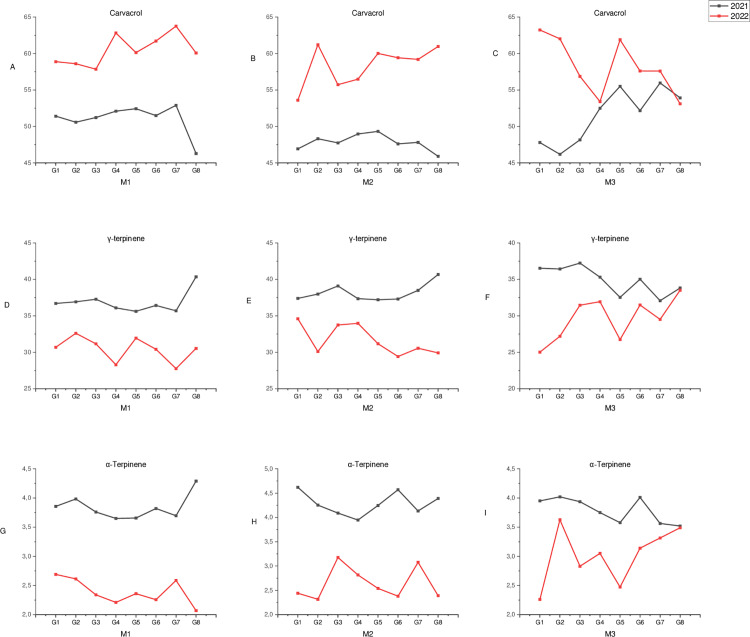
Changes in essential oil content of summer savory genotypes over the years at different harvest times.

Among the observed essential oil components, carvacrol, γ-terpinene and α-terpinene were detected in the highest amounts at all harvest times. The total ratio of these essential oils was determined to be between 90.2–91. 8% on average depending on the harvest times in 2021, and this ratio varied between 90.9–93.3% in 2022. Variations occurred in terms of the harvest times of these essential oils due to the difference among genotypes in 2021 and 2022 (Fig 3A-F). While the carvacrol ratios of the genotypes showed higher rates in M1 (beginning of flowering) and M3 (full bloom) according to the harvest times in 2021 while the changes were detected according to the genotypes in 2022. In terms of γ-terpinene and α-terpinene content, the highest levels were recorded at M2 period in 2021, while their concentrations varied among genotypes in 2022 (Fig 3A-F). When the essential oil content of summer savory genotypes was evaluated across the years (2021 and 2022), carvacrol had higher values at all three harvest times in 2022 compared to 2021, whereas γ-terpinene and α-terpinene were detected at higher levels in 2021 than in 2022 (Fig 4A-I).

## Discussion

This study resulted in several important findings regarding the effects of genotypes and harvest times of summer savory on the yield and quality characteristics of this medicinal plant. The results provided the specific harvest period in which these genotypes could be harvested to obtain high yields and essential oil contents. An increase in the height of the plant was observed at full bloom stage (M3) as the summer savory genotypes continued to grow and develop from the beginning of flowering to full bloom. Our results align with previous studies on different plants (*Origanum onites*, *Dracocephalum moldavica*), which reported that the highest plant height was reached at the full bloom stage [39,40]. The results of Danalou and Ozer) [41] also proved that the maximum plant height of summer savory genotypes were obtained in the Iranian genotype (40.8 cm), and the lowest plant height was obtained in the Turkiye genotype (29.4 cm). In addition, studies on the height of summer savory plants reported that the average height is between 10–35 cm [42] with 23.73–40.83 cm observed in Turkiye [43–45], 32–60 cm in Hungary [46] and 23.5–39.9 cm in Poland [47]. The similarity or difference of the results obtained in our study may be due differences in genotypic characteristics, ecological factors and cultural practices.

The most favorable harvest time for achieving the highest yield of summer savory was the full blooming period (M3) regardless of the genotype. This result is consistent with the study conducted by Ziombra and Franszczak [48] in Spain, reporting that the most suitable harvest time for the summer savory is full bloom; however, it is in contrast with the results reported by Skubijand Dzida [13] in Poland, reporting that the highest values for height were obtained from plants harvested at the first bloom stage. This difference may be due to the variations in the genotypes used, and the differences in the ecological and climatic factors of the experimental area. Many studies have found that fresh yield of summer savory differed among geographical areas and ranged between 7905–37653 kg ha^-1^ in Turkiye [45,49], 23380 kg ha^-1^ in Iran [50] and 14120–27270 kg ha^-1^ in Poland [13] while dry yield was ranged between 1256–9440 kg ha^-1^[49,51]. The yield of fresh herb was lower than the values stated in the previous studies of Pirzad et al. [50] and Skubij and Dzida [13] and these were in line with the studies of Aşcı [45] and Katar [49] who observed similar results for dry herb yield. The differences and similarities between the fresh and dry herb values are thought to be due to the differences in the geographical areas where the study was conducted and the genotypes of summer savory used in the study. In terms of dry leaf yield, the yield values obtained in the study (Table 3) are similar to the values ranged between 679–3584 kg ha^-1^, obtained by different researchers [42,52,53]. - According to the harvest time and genotypes, the yield values of fresh herb, dry herb and dry leaf were generally higher in the first year (2021) than in the second year (2022). This may be due to the higher average temperature in 2021 compared to 2022 during which the crop was grown (Table 1). The study conducted by Katar et al. [54] also revealed that summer savory grown in hot climates, produces higher yields.

Types of environmental stresses such as physical (drought, soil type and irrigation, light intensity and wind) and chemical stresses (salinity, pH, fertilization, chemical composition and toxins) also affect the quality and quantity of essential oils of medicinal and aromatic plants [55] Essential oils and biosynthetic activities in medicinal and aromatic plants are regulated by genetic factors [56]. Moreover, the formation and accumulation of active substances in these plants are also under the influence of environmental factors [57–59] Sváb and Hornok [60] stated that the secondary metabolites and essential oil contents are significantly affected by ecological conditions. They reported that the main conditions that affect the formation of secondary metabolites and essential oils were light, its quality and intensity, photoperiod, temperature, water, soil, altitude, wind, and some other organic and inorganic factors. The difference in volatile oil contents and yield in our results and those of in previous studies may be due to the difference in the origins of the plant material used, differences in cultivation techniques, and the variability in climate and soil conditions across the regions where the studies were conducted. These results were also varied depending on the time of cultivation, as changes in climate conditions from year to year influenced the plant’s volatile oil contents. In the second year (2022), the high amount of rainfall and low average temperatures during the growing season negatively affected and reduced the volatile oil rate and yield (Table 1) and these results are in line with Katar et al. [54]. On the other hand, the essential oil ratios obtained in our study (3.23–4.73%) were higher than those reported by Danalou [43] of 0.72–1.14%, Dinc [51] of 3.15%, Ozgen [61] of 1.53% and Khalid and Aisha [62] of 0.1–0.6%. However, these values fell within the range reported by other researchers as 1.66–4.64% by Hejja et al. [46], 1.28–4.75% by Baser et al. [28] and 2.52–5.59% by Skubij and Dzida [13]. In the studies of different researchers, it has been reported that the essential oil yield of summer savory varies between 0.61-1.12L ha^-1^ [51,62,63].

In the current study, carvacrol was determined as the main component and was present at the highest rate followed by γ-terpinene and α-terpinene during both the years. Carvacrol and γ-terpinene have been reported as the main components of summer savory genotypes by several researchers, worldwide [13,15,54]. The essential oil contents and ratios in aromatic plants are affected by the climatic factors, agronomic characteristics and genetic structure of the plant [64–66]. Increase in carvacrol levels were reported under stress conditions previously by Baher et al. [67]. In our study, the lower average temperatures in 2022compared to 2021 revealed that the higher carvacrol content observed may be a response to low temperature stress, which is one of the stress factors.

## Conclusion

Summer savory is one of the important medicinal and aromatic plants grown in many parts of the world, including Turkiye. The results of this study indicate that harvesting all summer savory genotypes at full blooming (M3) under the current climate change scenario is important for obtaining the highest yield of fresh and dry herb, dry leaf and essential oil. The results of the study also indicated that genotype G6 (North Macedonian origin) or G7 (Turkiye origin) can be recommended for fresh herb yield, depending on the climatic conditions. For dry herb, dry leaf and essential oil yield, genotype G7 (Turkiye origin) is recommend. In terms of essential oil content, it is recommended to harvest the plants at 40–60% blooming stage (M2). Overall, this study emphasizes the need to align harvesting strategies with genotype and climate conditions of a specific area.
